# Cost and utilization outcomes of pharmacist-led medication therapy optimization in a Medicare ACO population

**DOI:** 10.1093/haschl/qxag223

**Published:** 2026-08-26

**Authors:** Hazem F Elewa, Joseph F Levy, Chintan Pandya, Steven E Goldberg, Travis A Morgan

**Affiliations:** DecisionRx, Inc., Indianapolis, IN 46278, United States; Department of Health Policy and Management, Johns Hopkins Bloomberg School of Public Health, Baltimore, MD 21205, United States; Department of Health Policy and Management, Johns Hopkins Bloomberg School of Public Health, Baltimore, MD 21205, United States; DecisionRx, Inc., Indianapolis, IN 46278, United States; DecisionRx, Inc., Indianapolis, IN 46278, United States

**Keywords:** medication therapy optimization, pharmacogenomics, comprehensive medication management, difference-in-differences, Medicare, polypharmacy, value-based care

## Abstract

**Introduction:**

Medication-related problems are a persistent source of avoidable spending among Medicare beneficiaries, presenting an important opportunity to improve the quality of care for seniors and reduce financial burden on the Medicare program.

**Methods:**

We evaluated a pharmacist-led medication therapy optimization (MTO) program implemented in a Medicare accountable care organization population using a regression-adjusted difference-in-differences design with propensity score matching. The analysis included 1003 treated members and 14 031 matched controls contributing 334 531 member-months of claims data (2022-2025). The MTO program combined pharmacogenomic testing, a nine-vector medication-risk algorithm, comprehensive pharmacist review, and structured recommendations to prescribers.

**Results:**

MTO participation was associated with a statistically significant reduction in total medical costs (pooled average treatment effect on the treated: −19.4%, −$246 per member per month [PMPM]; *P* < 0.001). The treatment effect was strongly duration-dependent, growing from −4.7% in the first three months to −31.9% (−$462 PMPM; *P* < 0.001) beyond nine months of program benefit. All care settings showed significant reductions: inpatient admissions −43.1% (*P* < 0.001), emergency department visits −41.3% (*P* < 0.001), and outpatient visits −28.3% (*P* < 0.001).

**Conclusion:**

Medication therapy optimization was associated with significant cost savings. Parallel trends validation and a pronounced dose-response relationship support a causal interpretation. Short-duration evaluations are likely to substantially underestimate the full economic value of an MTO program.

Key Points

- A pharmacist-led medication therapy optimization program was associated with a 19.4% reduction in total medical spending (−$246 per member per month) among Medicare accountable care organization beneficiaries.
- Savings increased with program exposure, reaching 31.9% (−$462 per member per month) beyond nine months, consistent with a dose-response relationship.
- Spending reductions were concentrated in acute care, with inpatient admissions reduced 43.1% and emergency department visits reduced 41.3%.

## Introduction

Recent policy and clinical literature have increasingly framed polypharmacy as a chronic condition requiring structured longitudinal management rather than isolated episodic intervention.^1^ Contemporary analyses have further emphasized the importance of deprescribing frameworks and medication optimization strategies in older adults exposed to high medication burden.^2^ Medication-related care gaps are especially common in Medicare populations and are associated with decreased efficacy and increased risk of adverse drug events, drug-drug interactions, and avoidable healthcare utilization.^3^ These medication failures persist for myriad reasons, including patients’ unique genetics, lifestyle, and clinical history. The cost of non-optimized medication therapy in the United States comprised approximately 16% of all healthcare spending in 2016,^4^ far more than heart disease, diabetes, or cancer. A substantial portion of this waste is preventable through enhanced interdisciplinary care coordination and application of evidence-based prescribing guidelines.

Comprehensive medication management (CMM) programs, in which clinical pharmacists systematically review medication regimens and identify drug therapy problems, have demonstrated value in outpatient and integrated care settings.^5-7^ More recently, the integration of pharmacogenomic (PGx) testing into CMM has emerged as a promising strategy for optimizing drug selection and dosing based on individual genetic variations that impact drug safety and/or efficacy.^8,9^ The DecisionRx program evaluated herein is a PGx-enriched medication therapy optimization (MTO) model in which clinical pharmacists use broad-panel PGx test results, medication risk-scoring algorithms, and comprehensive patient data to generate standardized Medication Action Proposals (MAPs) with specific, actionable recommendations for prescribers.

Prior evaluations of similar interventions have demonstrated promising results.^10^ Jarvis et al.^11^ reported savings of 6.8% ($218 PMPM; *n* = 5288) in direct medical charges in a Medicare Advantage population over a 32-month observation period. El Rouby et al.^12^ reported an 8.1% net cost reduction ($132 in medical savings and $20 in pharmacy savings; *n* = 548) among a Medicare Advantage population receiving PGx testing within a primary care setting over 12 months. Fragala et al.^13^ found significant reductions in inpatient and emergency department utilization (−39% in both; *n* = 452) in a younger, employed population.

However, important evidence gaps remain. Two of these studies (Jarvis et al.^11^ and El Rouby et al.^12^) employed a difference-in-differences design but did not formally validate the parallel pre-treatment trends assumption that underwrites causal inference; Fragala et al.^13^ used propensity matching with doubly robust regression in a pre-post framework, foregoing a quasi-experimental temporal contrast altogether. None reported duration-stratified effect estimates that would reveal how the treatment effect evolves during exposure length. All were further limited by narrow PGx panels covering 21 to 27 pharmacogenes, a fraction of the actionable drug-gene variants currently documented in the clinical literature.^14^

This study extends this evidence base through a methodologically rigorous regression-adjusted difference-in-differences design, applied to a large propensity-score-matched Medicare ACO population. The primary outcome is a duration-stratified average treatment effect on the treated (ATT) for total medical cost per member per month (PMPM), which characterizes how savings accumulate over time and provides the primary evidence for a causal interpretation. Pre-specified secondary outcomes include healthcare resource utilization and cost by care setting. Exploratory heterogeneity analyses by baseline cost intensity, morbidity profile, and various clinical subgroups were used to generate hypotheses for future confirmatory work.

## Data and methods

### Study design and setting

This retrospective cohort study used a regression-adjusted difference-in-differences design, supported by pre-analysis propensity matching.^15^ The study population was drawn from Medicare ACO beneficiaries who were continuously attributed and observable in claims to the ACO from October 2022 through December 2025. Because MTO interventions occurred at different times, treated members were matched with never-treated controls indexed to the same calendar month, reducing concerns about the negative-weighting problem that affects staggered-adoption two-way fixed-effects estimators.^16^ The model is specified at the member-month panel level to properly weight heterogeneous exposure durations.^17^ Propensity score matching pre-balances treated and control cohorts on observed baseline characteristics; regression adjustment via baseline covariate × post-period interaction terms addresses residual imbalance on clinical complexity measures.^15^ Inference uses cluster-robust standard errors with bootstrap refinement,^18^ and the conditional parallel trends assumption is assessed through a pre-intervention event-study specification. All claims were filtered to a uniform 90-day maximum payment lag to mitigate bias from variation in adjudication timing.

### Intervention

The DecisionRx program is a pharmacist-led medication therapy optimization (MTO) service. ACO patients presenting with high polypharmacy risk (defined as scoring among the highest 50% of ACO beneficiaries using a standardized 9-vector polypharmacy risk score) were eligible for participation in the MTO program. Of 2765 invited high-risk patients, 1023 (37%) agreed to participate. Participation included completion of a brief intake form and in-home collection of a buccal sample for Pharmacogenomic testing by a CLIA-certified laboratory. Clinical pharmacists conducted comprehensive medication reviews for each patient, integrating test results from a broad PGx panel covering 48 pharmacogenes,^14^ medication risk-stratification algorithms, and patient-specific clinical data obtained from claims and patient consultation. Program outputs included a standardized Medication Action Proposal (MAP) with prioritized recommendations including dose adjustments, therapeutic substitutions, deprescribing/discontinuation, new medication initiation, and monitoring flags, which were communicated to patients and their prescribers to inform clinical decision-making.^19^ Pharmacists conducted live teleconsultation with patients and prescribers on a voluntary basis but did not modify prescriptions or dispense medications. All patient management was conducted by primary care providers or prescribing specialist care teams, with DecisionRx pharmacists acting as a supplemental clinical resource to those teams.

### Participants

The full analytic population comprised 97 639 members, of whom 1023 had received the MTO intervention. Enrolled patients were treated by 107 primary care providers practicing at 40 practice groups, aligned to two Medicare ACOs. Ninety-three percent of patients were taking five or more prescriptions, and 30% had filled prescriptions from three or more prescribers. Monthly polypharmacy risk scores were assigned to all beneficiaries using an algorithm comprising nine distinct medication failure vectors: pharmacogenetics, drug-drug interactions, therapeutic duplication, adverse effects, black box warnings, and contraindications with disease, lifestyle, age, and maternity. Study eligibility required (a) enrollment continuity inferred by at least one incurred claim prior to 12 months before the index date (the date of MAP generation for the patient) and at least one claim during the final 3 months of the evaluation period, and (b) at least three active ingredients prescribed during the 12 months preceding the index date. After applying eligibility criteria, 1005 treated members and 40 406 eligible controls remained (see Appendix Figure AS1).

### Detrending

Prior to baseline construction and propensity score matching, we removed secular cost trends and seasonal variation from the claims data by regressing monthly total costs (after Winsorization at the 99.8^th^ percentile) on a linear calendar-time index and calendar-month indicator variables. This model was estimated exclusively on control-group observations, then applied to all rows in the dataset. The resulting residuals—actual cost minus predicted calendar-time trend—served as the basis for all downstream cost measures. Performing detrending upstream of matching ensures that baseline cost covariates are purged of inflation and seasonality, eliminating calendar-time confounding inherent to the study's staggered enrollment.

### Matching procedure

A propensity score model was estimated via logistic regression with covariates including age, sex, and assorted baseline cost and ACG® System^20^ care intensity variables. Treated members were matched with up to 20 non-treated controls using month-constrained nearest-neighbor matching (caliper = 0.05 SD of the logit propensity score, without replacement). Matched controls inherited the intervention date of their nearest treated member and were screened for continuous enrollment based on the inherited index date, yielding a final analytic dataset of 1003 treated and 14 031 controls, contributing 334 531 member-months of claims exposure. Baseline covariates for both arms are measured over the 12-month window ending one month before the index date to which the member is matched, so that intervention and control covariates share a common reference period.

### Study population and balance

Table 1 presents baseline covariate distributions and standardized mean differences (SMDs) for the matched cohort. Of 13 covariates, 12 achieved a |SMD|<0.10 after matching, indicating adequate balance.^21^ The single covariate with residual imbalance (active ingredients, SMD = 0.106) reflects known clinical complexity differences between the high-polypharmacy population targeted for MTO and the broader Medicare beneficiary pool. All 13 covariates are included with post-period interaction terms in the outcome regression to account for this residual confounding.

**Table 1.** Baseline characteristics of matched cohorts.

Characteristic	Intervention (*n* = 1003)	Control (*n* = 14 031)	SMD
Female, *n* (%)	559 (55.7%)	7807 (55.6%)	0.002
Age, mean (SD)	75.3 (8.3)	75.2 (8.9)	0.010
Malignancy, *n* (%)	437 (43.6%)	6025 (42.9%)	0.013
ER visits (per 1k MM)	41.5 (81.9)	42.9 (92.4)	−0.016
IP admissions^a^	16.0 (55.1)	15.6 (50.3)	0.008
IP days^a^	80.2 (388.2)	76.3 (344.9)	0.011
OP visits^a^	691.6 (1115.8)	715.7 (1205.4)	−0.021
PMPM, mean (SD)	$903.76 ($1440.91)	$922.12 ($1486.72)	−0.013
Cost slope, mean (SD)	$3.83 ($287.13)	$14.07 ($287.69)	−0.036
Concurrent risk, mean (SD)	5.9 (3.9)	5.8 (3.9)	0.022
Active ingredients, mean (SD)	14.6 (7.5)	13.7 (8.0)	0.106
Frailty flag, n (%)	369 (36.8%)	5049 (36.0%)	0.017
Chronic conditions, mean (SD)	13.9 (6.4)	13.4 (6.5)	0.071

Source: Authors’ analysis of ACO claims data.

^a^Per 1000 member-months. MM, member months; SD, standard deviation; SMD, standardized mean difference; PMPM, per member per month; ED, emergency department; IP, inpatient; OP, outpatient; Concurrent Risk, ACG® metric that models the relative resource utilization for individuals (R-squared for elderly populations ranges from 0.473-0.546); Chronic Conditions, ACG® metric defined as the number of concurrent conditions likely to last longer than 12 months and negatively impact health or functional status. All 13 covariates enter the outcome regression with post-period interaction terms regardless of SMD.

### Parallel trends validation

**Figure 1.**
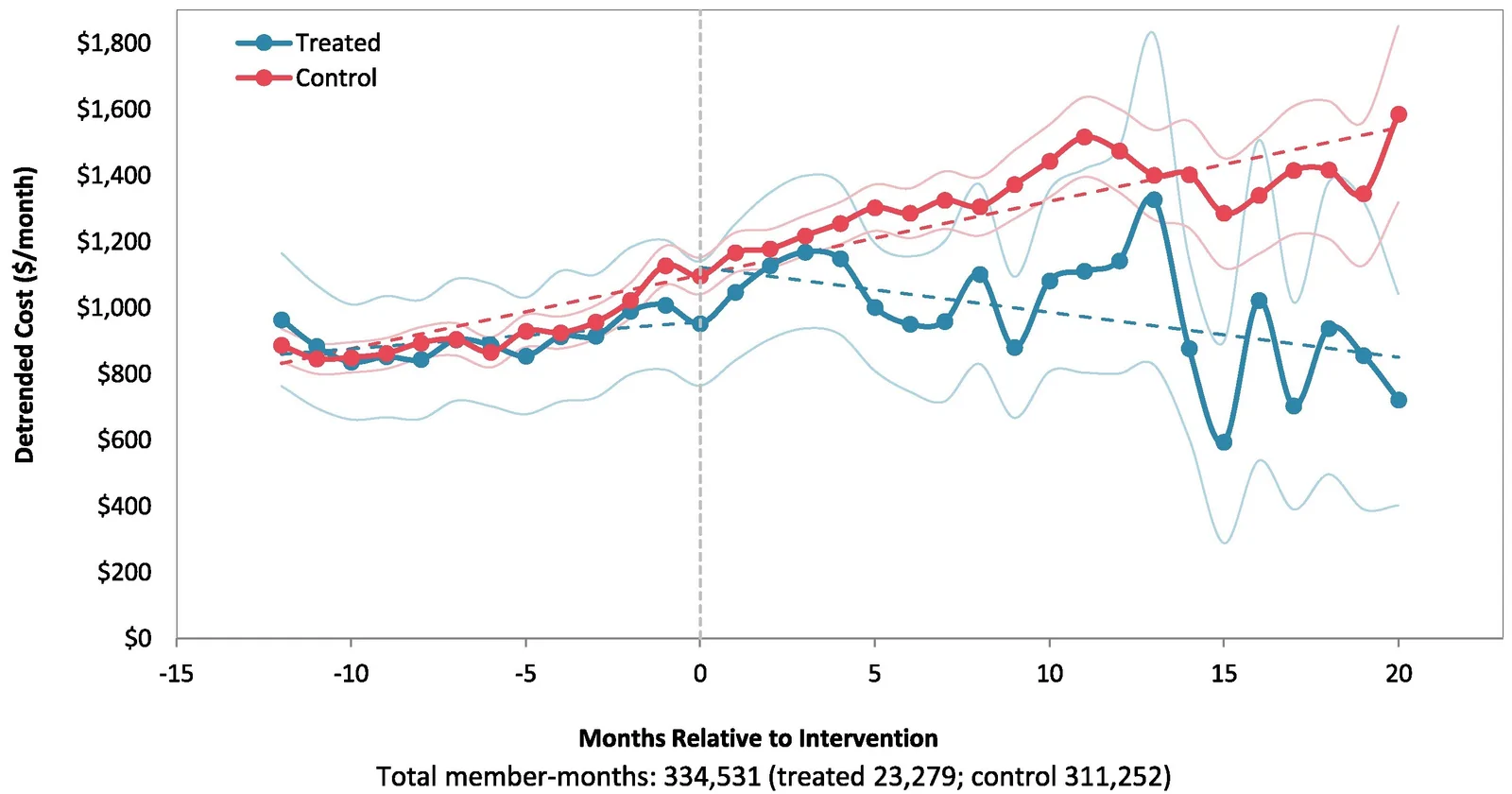
Event Study of Detrended Monthly Costs (Parallel Trends Validation). Source: Authors’ analysis of ACO claims data. Series are unadjusted mean detrended cost per member-month for matched treated (*n* = 1003) and matched control (*n* = 14 031) members. Vertical dashed line = intervention month (0). Pre-intervention cost trajectories are approximately parallel (joint F-test on unadjusted pre-period event-time × treatment interactions: *F*(11 180 384) = 0.299; *P* = 0.986). Post-intervention, treated costs diverge downward from controls. Bounded regions = 95% CI around each arm mean. Truncated at event-times with *N* ≥ 100 in both arms.

The formal parallel trends test yielded a non-significant joint F-test of pre-period event-study coefficients (*F*[11 180 384] = 0.299; *P* = 0.986), consistent with the assumption of parallel pre-intervention cost trajectories. Pre-intervention detrended cost slopes were $3.83/month for the MTO group and $14.07/month for the control group (SMD = −0.036), a negligible difference relative to within-group standard deviations of approximately $280/month. Figure 1 provides visual confirmation of similar pre-intervention trends.

### Outcomes

The primary outcome was duration-stratified ATT on medical cost PMPM, estimated on detrended data. Pre-specified secondary outcomes were healthcare resource utilization and cost by service category (emergency department, inpatient, outpatient, and professional services). Exploratory heterogeneity analyses were conducted by baseline demographic and care intensity metrics and are presented in the Appendix.

### Statistical analysis

The ATT was estimated via panel-level (member-month) regression-adjusted difference-in-differences (pooled OLS) on detrended costs, with all propensity score covariates included as level and post-period interaction terms (subject to VIF<10 screening) to absorb residual confounding. Standard errors were clustered at the member level. For the pooled estimate (G > 5000 clusters), *P*-values were computed from the standard normal distribution under H_0_: ATT = 0 (two-sided); subgroup estimates used the t(G−1) distribution to account for smaller cluster counts.^18^

### Ethical approval and consent

The study was determined to be exempt from human subjects review because it involved secondary analysis of existing de-identified data. Informed consent was not required.

## Results

### Pharmacist recommendations

In aggregate, 914 patients in the intervention cohort (91%) received at least one actionable pharmacist recommendation, totaling 3312 recommendations (mean: 3.6 recommendations per patient). No therapy modifications were recommended for the remaining 89 intervention patients. Among patients receiving actionable recommendations, 66% were advised to consider changing at least one medication, with the remaining 34% advised to closely monitor a specific medication failure metric. The distribution of medication change recommendations was 58% deprescribing, 27% therapeutic substitution, 12% dose modification, and 3% initiation of a new drug (Appendix Figure AS2). Approximately one-third of all pharmacist recommendations were driven by pharmacogenomic risk, while two-thirds related to the eight additional risk vectors comprising the polypharmacy risk score. The drug groups most commonly flagged for therapeutic replacement or discontinuation were antihyperlipidemics, hematological agents, antidepressants, antihypertensives, anticholinergics, and analgesics. Subsequent prescriber adoption of recommendations was monitored via ongoing monthly claims surveillance and polypharmacy risk scoring (see Exploratory Analysis below).

### Duration-stratified dose response

**Figure 2.**
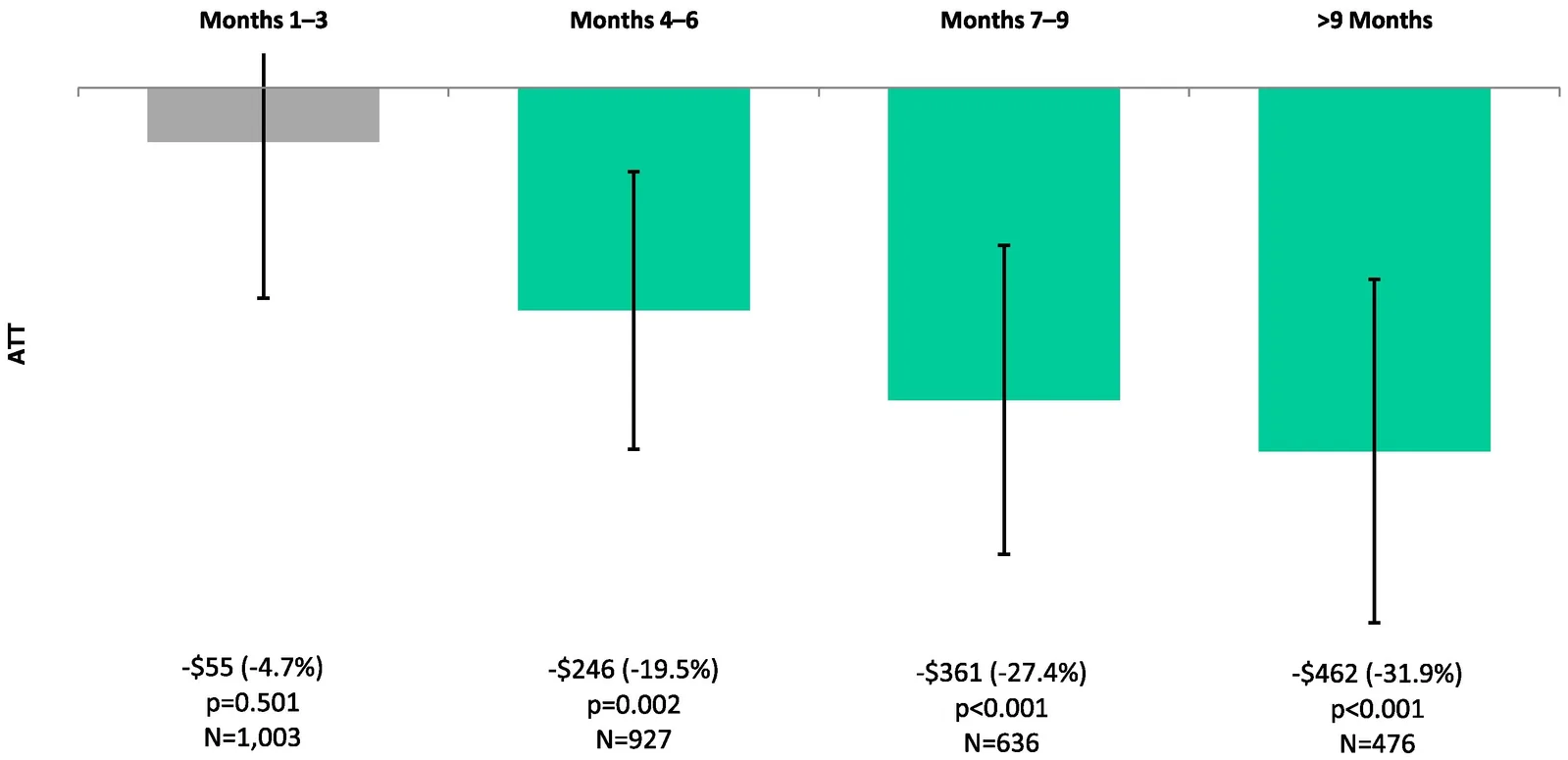
Treatment Effect by Duration Since Intervention (Dose-Response Curve). Source: Authors’ analysis of ACO claims data. Monotonic dose-response: −4.7% at months 1-3 (−$55 PMPM; *P* = 0.501), escalating to −31.9% at >9 months (−$462 PMPM; *P* < 0.001). Gray bar indicates non-significant window; green bars indicate statistically significant windows. Error bars = 95% confidence interval.

Among 10 240 post-treatment member-months, the MTO program was associated with a statistically significant reduction in detrended total medical costs by 19.4% (−$246 PMPM; *P* < 0.001). The ATT exhibited a strong monotonic dose-response relationship with follow-up duration (Figure 2), constituting the primary evidence for a causal interpretation.

During the initial 1-3 months following intervention, the estimated ATT was −4.7% (−$55 PMPM; *P* = 0.501), a directional but not statistically significant reduction, consistent with the ramp-up phase immediately following MAP delivery during which prescribers are reviewing recommendations and regimen changes are only beginning to be implemented. Effects grew substantially thereafter, reaching −19.5% (−$246 PMPM; *P* = 0.002) at months 4-6, −27.4% (−$361 PMPM; *P* < 0.001) at months 7-9, and −31.9% (−$462 PMPM; *P* < 0.001) beyond 9 months of intervention benefit. This progressively increasing effect size is inconsistent with regression to the mean or transient care-coordination effects, both of which would be expected to attenuate rather than increase over time.

### Cost and utilization by care setting

Cost reductions were concentrated in acute and facility-based settings. Inpatient costs showed the largest absolute reduction (−$101 PMPM; *P* < 0.001), followed by outpatient (−$101 PMPM; *P* < 0.001) and emergency department (−$10 PMPM; *P* < 0.001), all reflecting savings of approximately 25%-31% relative to counterfactual spending. Professional fees showed a modest directional reduction that did not reach significance (*P* = 0.065), consistent with the expectation that the intervention's primary mechanism operates through reductions in downstream acute care rather than changes in physician encounter volume. Pharmacy cost data were not available because Medicare Part D expenditures are not paid by the ACOs comprising this study. Detailed cost and utilization estimates by service category are presented in Table 2.

**Table 2.** Healthcare resource utilization and cost outcomes by service category.

	Frequency ATT			Cost ATT		
Service	Per 1k MM	% Effect	*P*-Value	$ PMPM	% Effect	*P*-value
Total cost	—	—	—	−$246	−19.4%	<0.001
ED	−17	−41.3%	<0.001	−$10	−28.2%	<0.001
IP (admissions)	−7	−43.1%	<0.001	−$101	−31.0%	<0.001
IP (days)	−51	−66.1%	<0.001			
OP	−198	−28.3%	<0.001	−$101	−24.7%	<0.001
Professional	—	—	—	−$31	−6.7%	0.065

Source: Authors’ analysis of ACO claims data.

MM, member-months; ED, emergency department; IP, inpatient; OP, outpatient.

The utilization results corroborate the cost findings. The largest relative reductions appeared in inpatient days (−66.1%; *P* < 0.001) and inpatient admissions (−43.1%; *P* < 0.001), suggesting that MTO may reduce both the likelihood of hospitalization and its severity when hospitalization occurred. Emergency department visits declined by 41.3% (*P* < 0.001), consistent with a reduction in acute medication-related events. Outpatient visits also decreased significantly (−28.3%; *P* < 0.001), which may reflect a reduction in follow-up encounters for medication-related complications rather than withdrawal from routine care.

### Exploratory analyses

The following analyses are exploratory and hypothesis-generating in nature; all qualifying subgroups are reported regardless of statistical significance. The strongest treatment effects were concentrated among members in the highest baseline cost-intensity quintile (−31.9%; *P* < 0.001) and highest clinical risk quintile (−25.1%; *P* = 0.001). Analysis by ACG® Resource Utilization Bands, which combine concurrent relative resource use and number of comorbidities, showed the greatest cost reductions in the very high utilization (RUB 5) stratum (−21.9%; *P* < 0.001). Savings were persistent across all sufficiently powered age groups. Significant cost reductions were observed among members without active malignancy (−26.0%; *P* < 0.001), while the malignancy sub-cohort showed a directional but non-significant effect (−9.9%; *P* = 0.172), a clinically expected pattern given that oncology treatment costs are driven by disease protocols rather than the polypharmacy targets of MTO. Patients with confirmed polypharmacy risk reduction post-intervention achieved larger savings (−18.2%; *P* = 0.087) than those without observed risk reduction (−15.6%; *P* = 0.001). Patients receiving recommendations with lower PGx risk were more likely to exhibit reduced PGx risk 3 months post-intervention than matched controls (36.0% vs 27.9%, *P* = 0.057). Patients with confirmed PGx risk reduction post-intervention showed a directionally larger but non-significant effect (−20.2%; *P* = 0.198) relative to those without observed risk reduction (−17.5%; *P* = 0.009).

The pharmacist intervention accelerated medication risk mitigation, providing potential mechanistic evidence linking the program to its downstream cost outcome. By 12 months post-intervention, 53% of treated patients who received risk-lowering MAP recommendations had achieved a lower total polypharmacy risk score than their intervention-month baseline, compared with 40% of matched controls with available risk-score data (Holm-corrected *P* = 0.004). The control group's natural rate of risk reduction reflects the trial-and-error medication management that occurs without explicit pharmacist oversight; the gap between cohorts is consistent with incremental optimization associated with the MTO intervention. Notably, this mechanistic gap is statistically significant at every post-intervention checkpoint (months 3, 6, 9, and 12) and widens over time, mirroring the cost-trajectory dose-response and reinforcing a causal interpretation grounded in the program's therapeutic logic. Detailed subgroup results are presented in the Appendix.

## Discussion

In this retrospective analysis of a Medicare ACO population, the pharmacist-led MTO program was associated with a statistically significant reduction in total medical costs, with significant savings across emergency department, inpatient, and outpatient cost subcategories and healthcare resource utilization frequency measures. The regression-adjusted specification combining propensity score matching with covariate × post interaction regression adjustment provides a conservative, methodologically rigorous estimate.

The most notable finding was the strong duration-response relationship: the regression-adjusted estimate of PMPM savings beyond 9 months of intervention benefit (31.9%) was nearly seven times the initial 1-3 month estimate (4.7%), and the initial window itself did not reach statistical significance. This pattern is entirely consistent with the program's therapeutic mechanism: changes to complex medication regimens unfold over months as pharmacist recommendations are reviewed by prescribers, regimen changes are implemented, and reductions in downstream medication-related events compound progressively. The implication for program evaluation is that analyses limited to short follow-up windows will not only underestimate the full clinical and economic value of MTO but may fail to detect any potential effect at all.

The magnitude and pattern of effects are broadly consistent with prior evaluations. Jarvis et al.^11^ reported 6.8% savings indexed to overall program implementation rather than patient-specific duration. Fragala et al.^13^ found significant reductions in inpatient and emergency department utilization in a younger employed population. The present study extends this evidence through a more rigorous propensity-score-matched regression-adjusted difference-in-differences design with formal parallel trends testing and time-stratified analysis.

Stratification results strengthen the causal interpretation by demonstrating potential mechanism specificity: absolute savings were largest in subpopulations expected to be more clinically affected, that is high-risk, high-cost, non-malignancy patients, as well as patients with observed post-intervention medication-risk reduction—while members outside of these subpopulations show notably smaller and less precise estimated effects. El Rouby et al.^12^ observed a substantially larger cost reduction among patients in whom medication changes were attributable to PGx guidance vs the overall savings estimate. Our study reinforces this finding by showing that adoption of broad pharmacist guidance, and PGx guidance in particular, are each associated with reduced polypharmacy risk and lower costs.

### Policy implications

The dose-response gradient observed here has direct implications for how pharmacist-led MTO programs should be evaluated and structured within value-based care arrangements.^22^ First, conventional payer evaluation windows, often three to six months, will systematically underestimate program value or fail to detect savings at all, as our months 1-3 result (*P* = 0.501) illustrates. Health plans and ACOs that contract for MTO services on a short-horizon return-on-investment basis are likely to undervalue these programs and underinvest accordingly; gainshare arrangements should be structured to measure savings over a minimum 9- to 12-month observation window.^23^ Second, the heterogeneity results suggest that program targeting matters. Treatment effects concentrate in the highest baseline cost and clinical risk quintiles, where savings of −$533 to −$842 PMPM are realized among members in the top fifth of either distribution; programs deployed to lower-acuity Medicare populations should expect more modest returns, consistent with the program's mechanism of reducing acute events potentially driven by polypharmacy. Third, the absence of significant savings among patients with active malignancy reflects the limits of medication optimization in populations whose costs are dominated by disease-specific protocols rather than polypharmacy risk; excluding active-oncology patients (or specific subsets thereof) from return-on-investment projections may be appropriate.

The savings measured here reflect the gross reduction in medical claims paid by the Medicare program, without any reduction for MTO program costs, which were funded entirely by the ACO sponsors under value-based care arrangements. No medical or pharmacy claims were submitted to Medicare for the PGx testing or pharmacist interpretation and consultation comprising the MTO program. Impact on paid pharmacy claims was not evaluated because Part D coverage is not attributed to ACOs in the Medicare Shared Savings or REACH programs, an area that warrants future research focus.

## Limitations

First, as a retrospective observational study, the design cannot definitively establish causation. However, the parallel trends validation (*P* = 0.986), monotonic dose-response, regression-adjusted estimation, and clinically coherent stratification collectively provide substantial support for a causal interpretation. Second, the sample size (*n* = 1003) provides adequate power for the primary analysis but limits precision for certain subgroup estimates; results will be updated as additional patients are enrolled and longer follow-up accrues. Third, the analysis does not capture pharmacy costs, which may increase if prescribers shift to more appropriate but costlier medications; prior evidence suggests any pharmacy cost increases are modest relative to medical savings. Fourth, the parallel trends test has limited power with monthly-level data and 12 pre-intervention periods; the event study plot (Figure 1) provides visual corroboration. Fifth, the continuous enrollment study design inherently introduces survivor bias, preventing measurement of effects during end-of-life care settings. Sixth, effects were estimated for the entire intervention cohort, regardless of prescriber adoption of pharmacist recommendations, and may understate the effect among patients for whom recommendations were implemented. Estimation by prescriber uptake warrants further study as additional data accrue. Seventh, results are derived from two Medicare ACOs and may not generalize to all Medicare populations.

## Conclusion

In a Medicare ACO population, a pharmacist-led MTO program was associated with a statistically significant reduction in total medical costs, with savings increasing monotonically as the duration of intervention benefit increased. The initial three-month window did not reach statistical significance, consistent with a controlled implementation ramp-up, while effects grew to over 30% by the ninth month and beyond. These findings support the clinical and economic value of pharmacist-led medication therapy optimization, informed by broad-panel PGx testing. The pronounced time-window gradient suggests that the full economic benefit of MTO is substantially larger than what can be captured in short-duration evaluations. The regression-adjusted design, formal parallel trends validation, and clinically coherent patterns of treatment effect heterogeneity strengthen the causal interpretation of these findings.

## Supplementary Material

qxag223_Supplementary_Data
