# Supplementary material for: Cost and utilization outcomes of pharmacist-led medication therapy optimization in a Medicare ACO population
Source: Health Aff Sch. 2026 Aug 26;4(9):qxag223. doi: 10.1093/haschl/qxag223 (PMC13602420; doi:10.1093/haschl/qxag223)
Supplement: Supplementary file 1 [file qxag223_supplementary_data.zip › Appendix_HealthAffairsScholar.pdf]

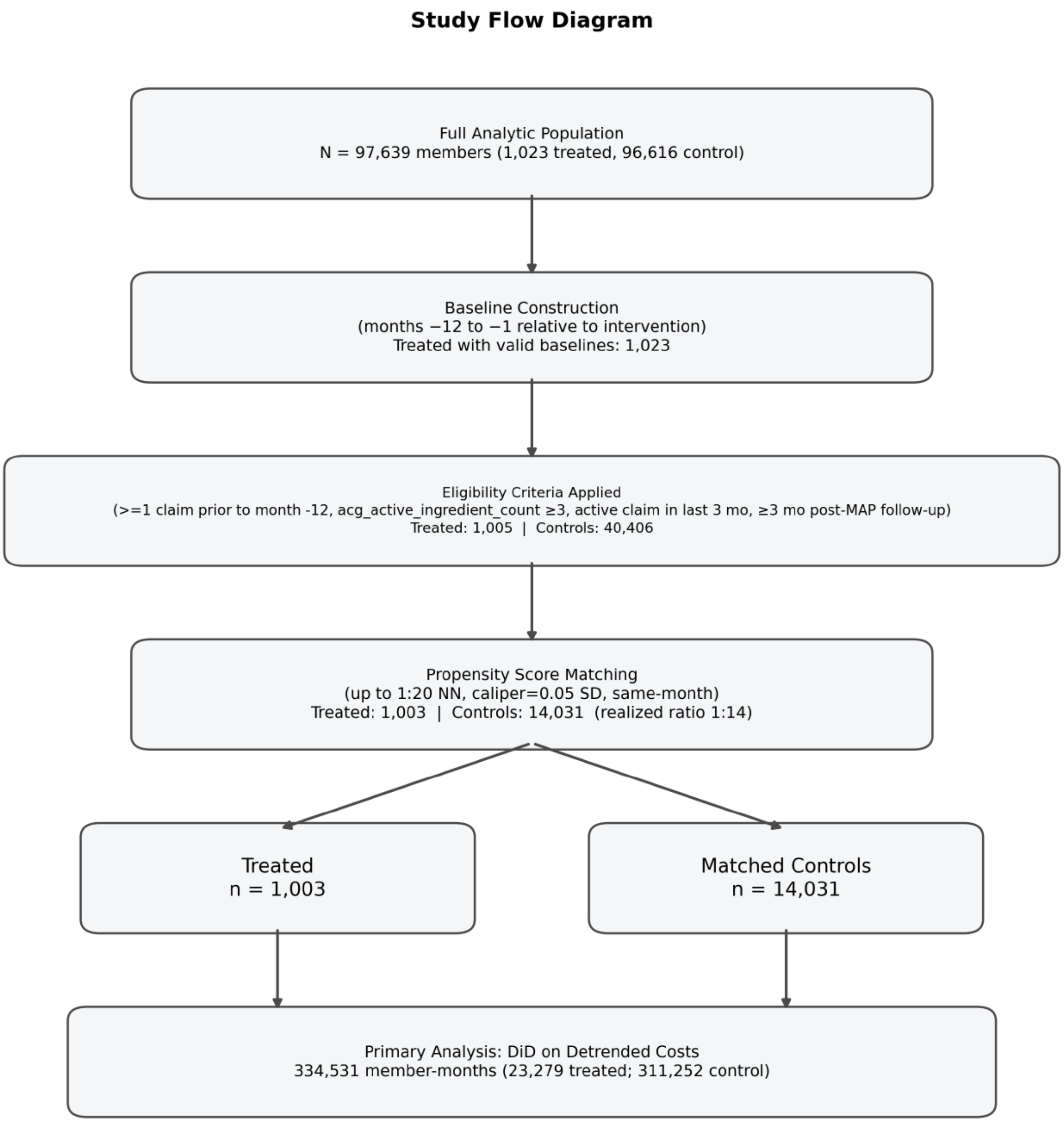

Figure A1. From 97,639 total beneficiaries (1,023 treated, 96,616 control), eligibility criteria (prior claim, ≥3 active ingredients, ≥3 months post-MAP follow-up) yielded 1,005 treated and 40,406 eligible controls. Propensity score matching produced a final analytic sample of 1,003 treated and 14,031 controls (334,531 member-months; 23,279 treated; 311,252 control).

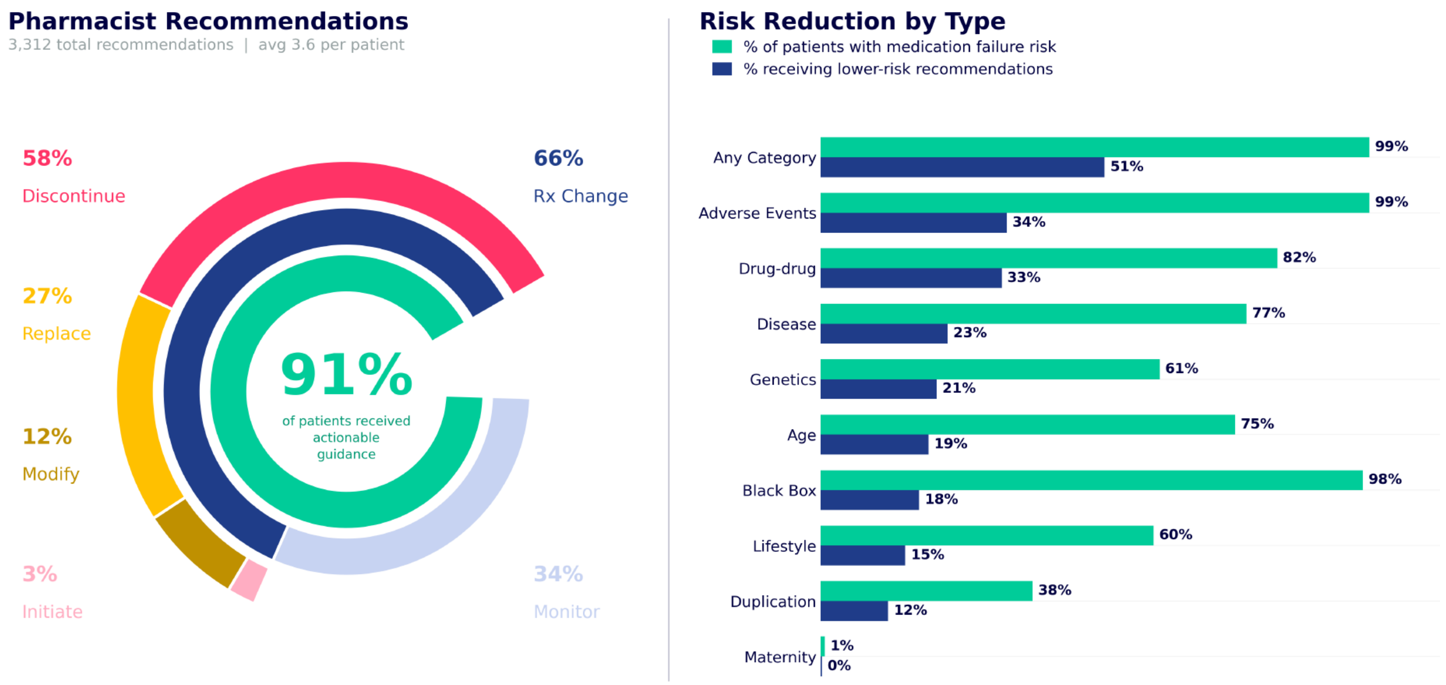

Figure A2. 91% of patients received actionable guidance; 3,312 total recommendations (avg 3.6 per patient). Recommendation type distribution: 58% deprescribing, 27% therapeutic substitution, 12% dose modification, 3% initiation. Risk reduction bar chart shows pre/post intervention medication failure prevalence across nine risk vectors.

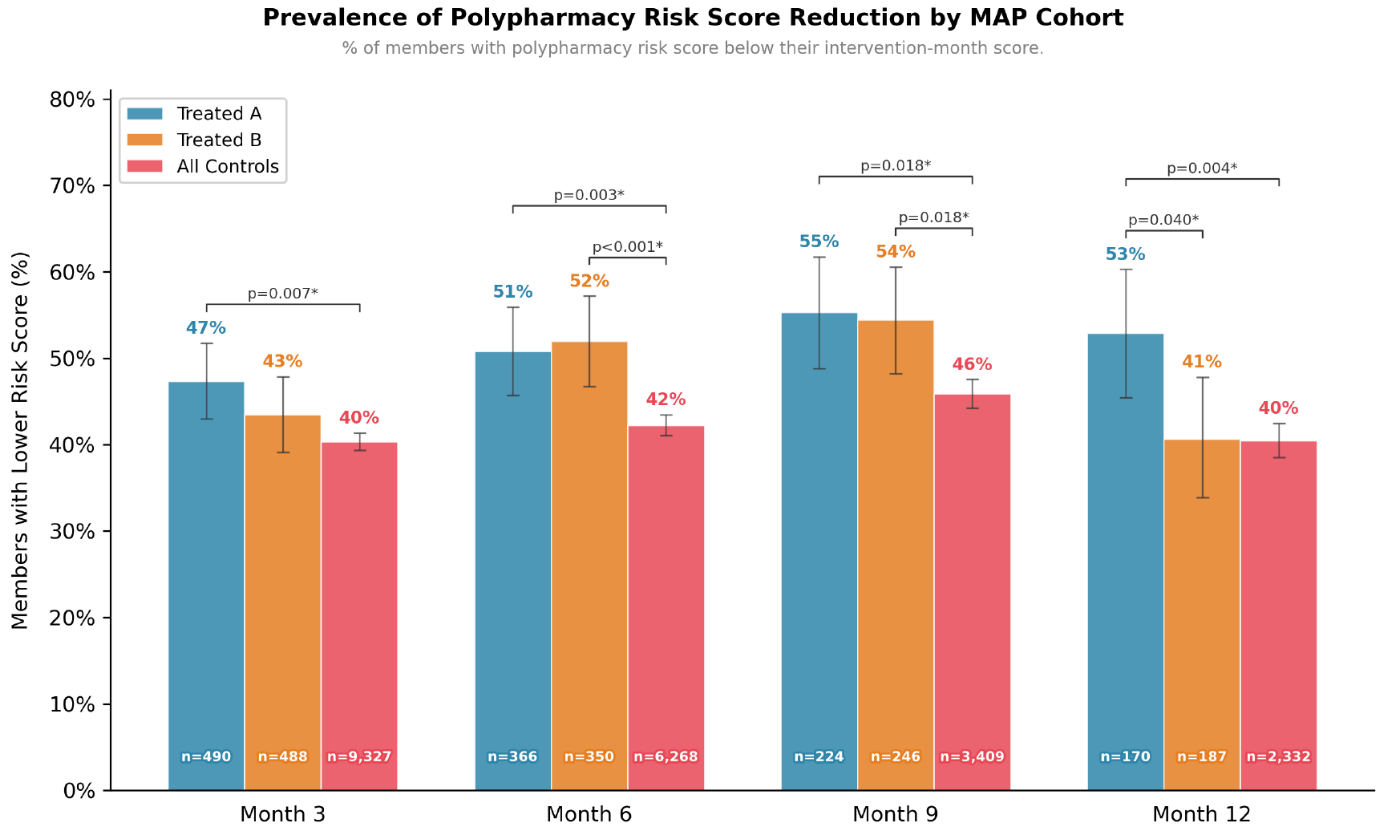

Figure A3. Percentage of patients with a total polypharmacy risk score below their intervention-month score at 3, 6, 9, and 12 months post-intervention. Treated A cohort is all patients who received risk-lowering MAP recommendations. Treated B cohort is all patients receiving a MAP that did <u>not</u> reduce total risk (though they may have received monitoring or equivalent-risk regimen modification recommendations). Error bars indicate Wilson 95% confidence intervals. Significance brackets denote Holm-corrected pairwise two-proportion z-tests.

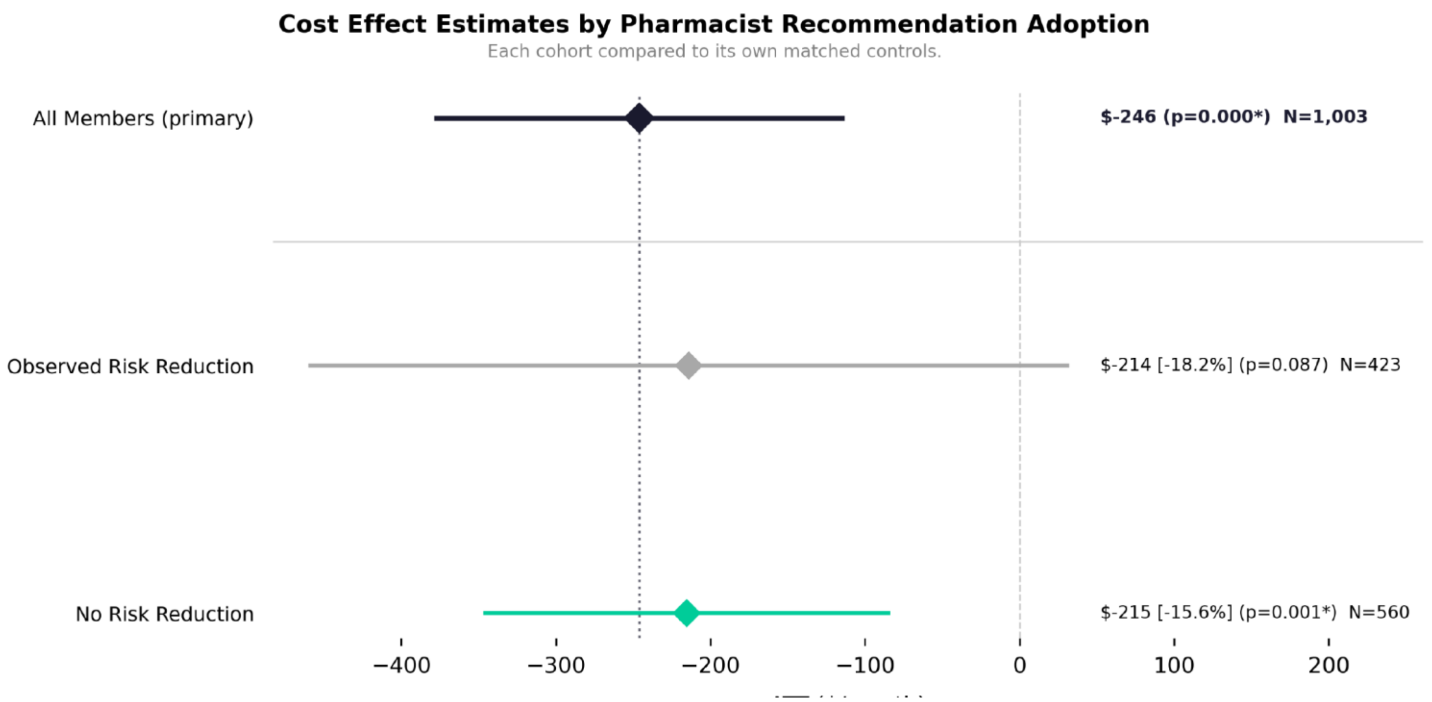

Figure A4. Forest plot of ATT estimates stratified by pharmacist recommendation adoption: Observed Risk Reduction (lower polypharmacy risk at last observed post-period month) and No Risk Reduction (observable risk data, no decrease). Each cohort compared to its own matched controls. 95% confidence intervals; *p<0.05.

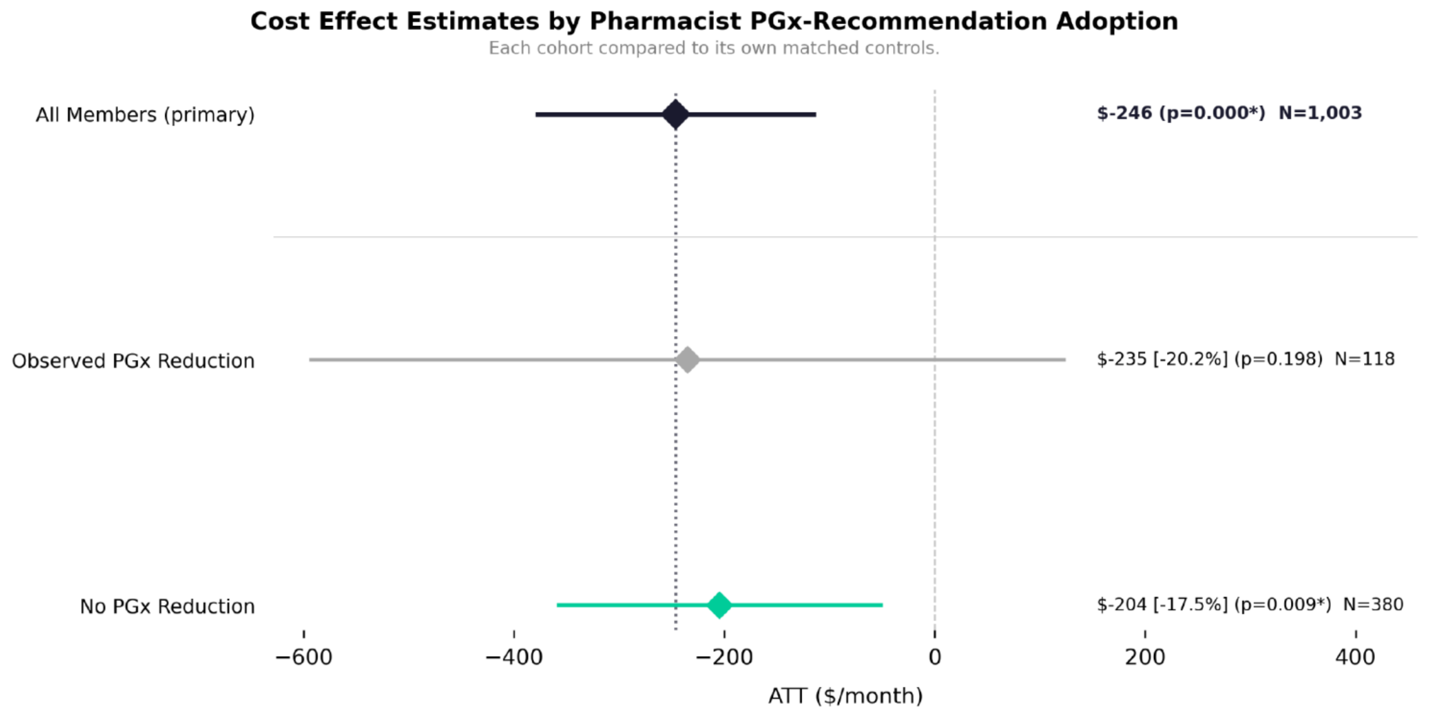

Figure A5. Forest plot of ATT estimates stratified by pharmacist PGx-recommendation adoption: Observed PGx Reduction (lower pharmacogenomic score at last observed post-period month) and No PGx Reduction (observable PGx data, no decrease). Each cohort compared to its own matched controls. 95% confidence intervals; *p<0.05.

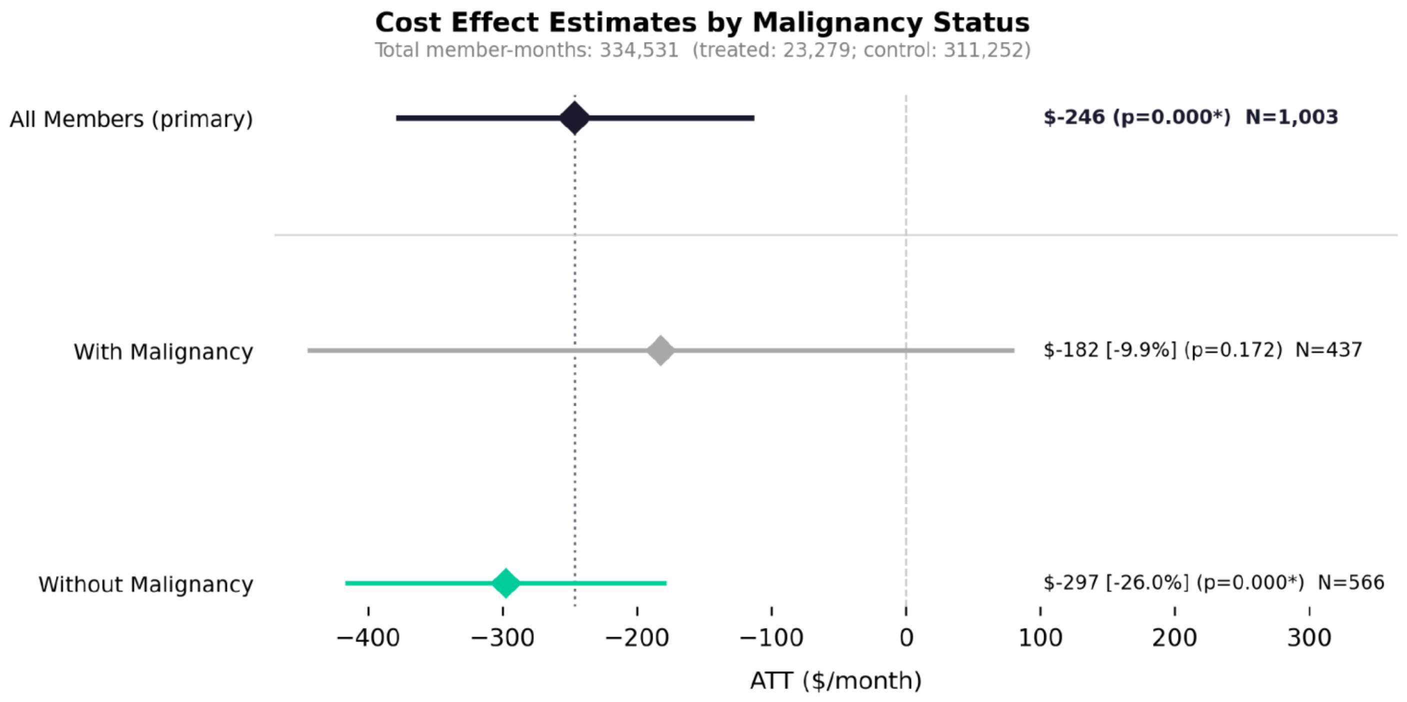

Figure A6. Forest plot of ATT estimates for the primary analysis and malignancy-stratified subgroups with 95% confidence intervals; *p<0.05.

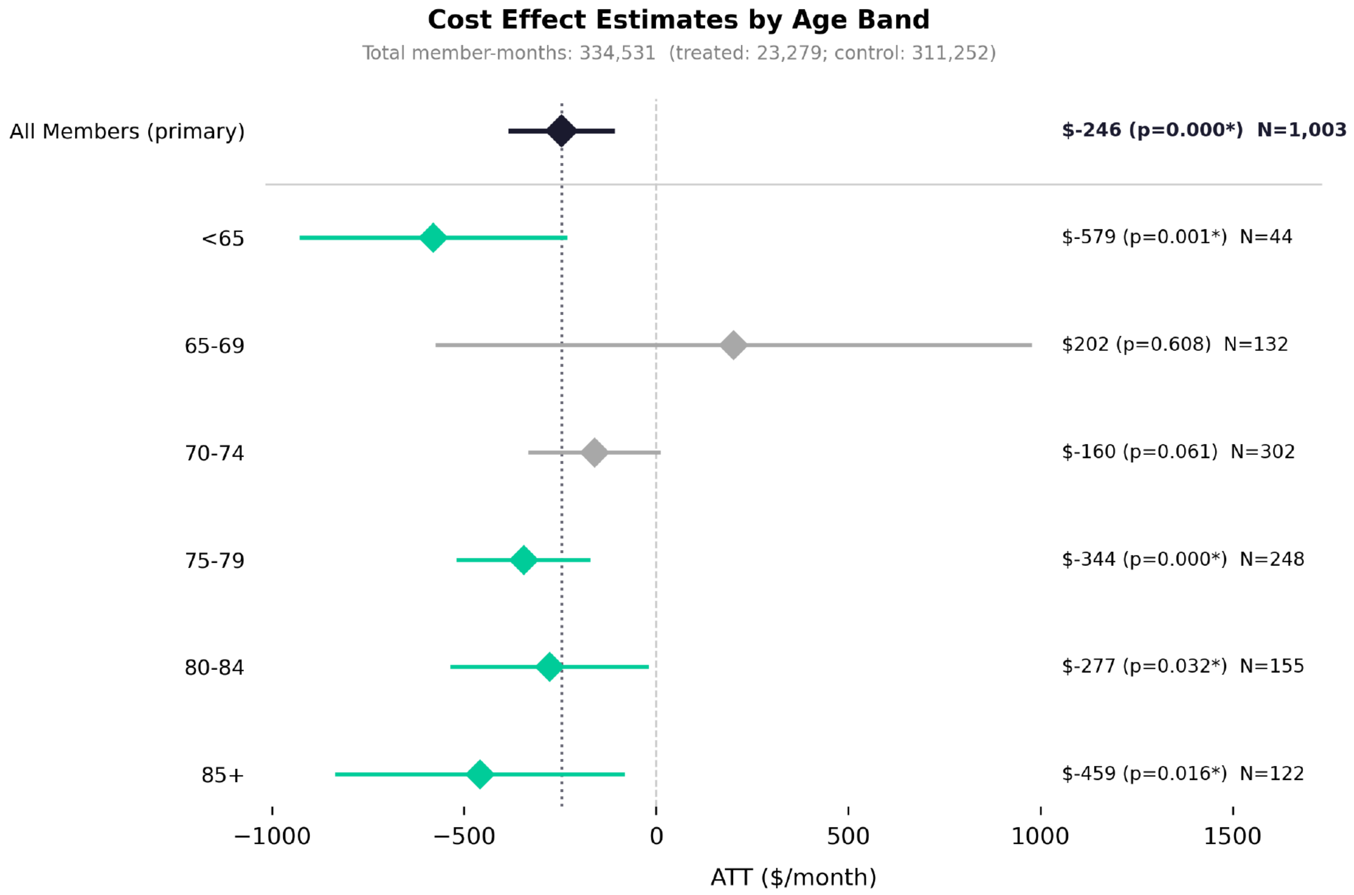

Figure A7. Forest plot of ATT estimates by age band with 95% confidence intervals; *p<0.05.

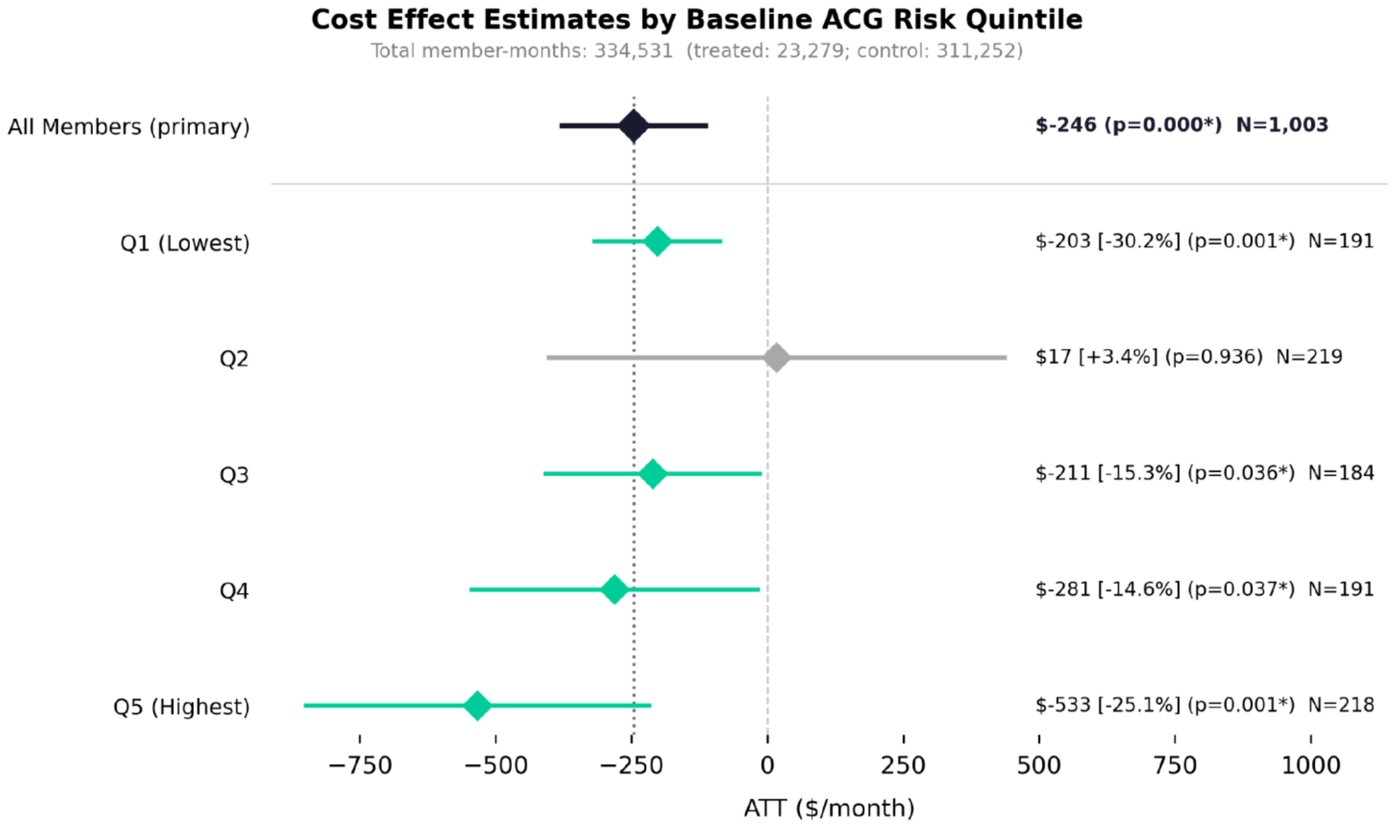

Figure A8. Forest plot of ATT estimates by baseline ACG concurrent risk quintile with 95% confidence intervals; *p<0.05.

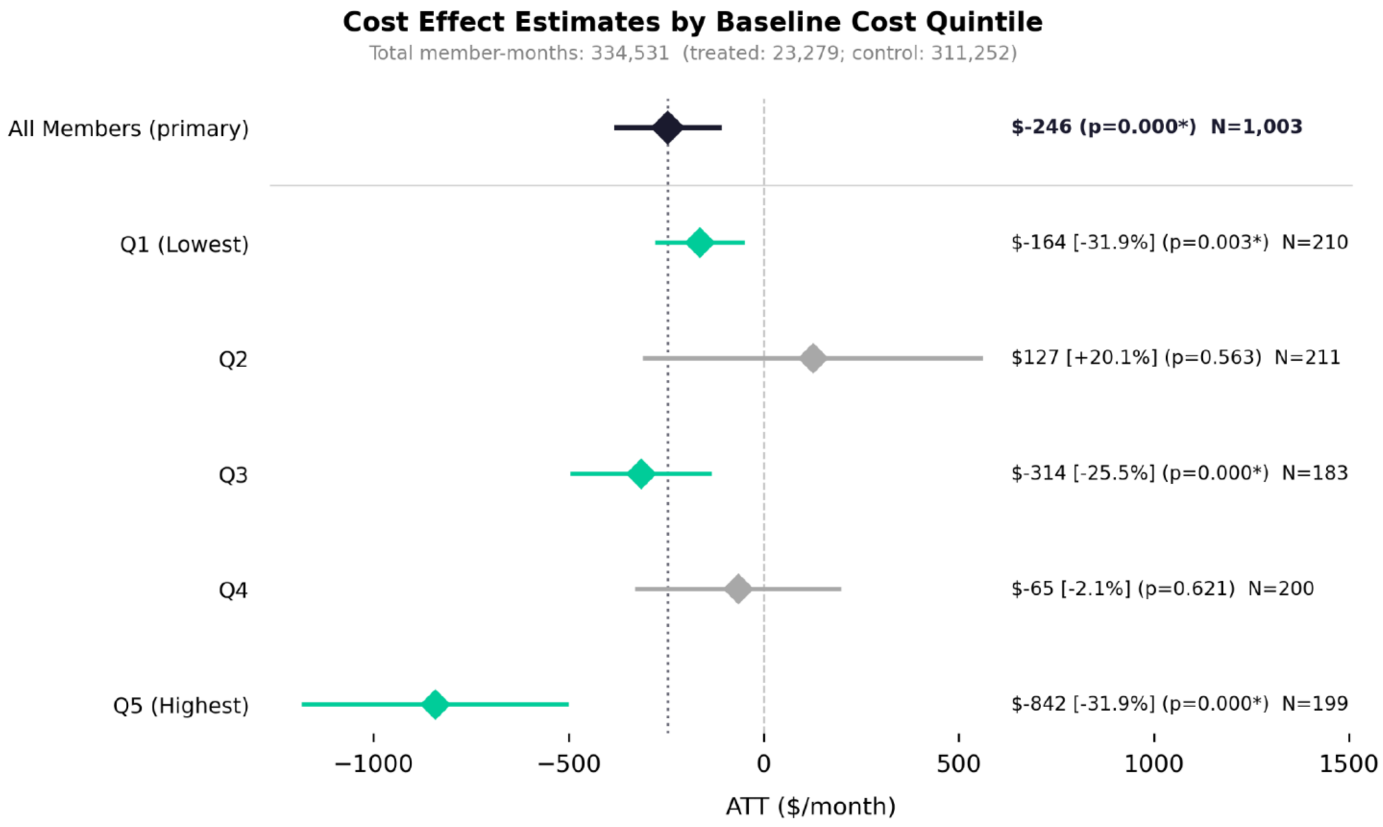

Figure A9. Forest plot of ATT estimates by baseline PMPM cost quintile with 95% confidence intervals; *p<0.05.

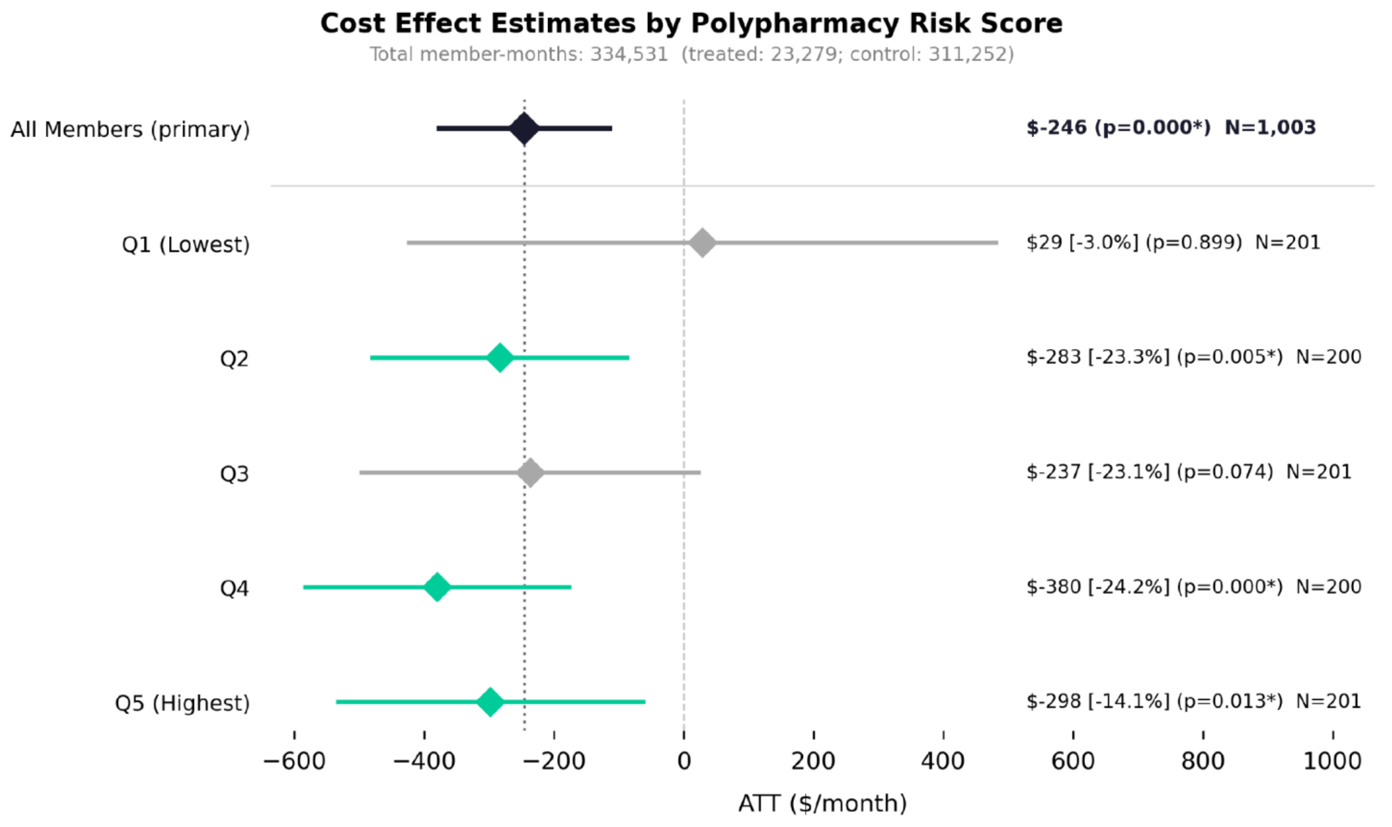

Figure A10. Forest plot of ATT estimates by baseline polypharmacy risk score quintiles with 95% confidence intervals; *p<0.05.

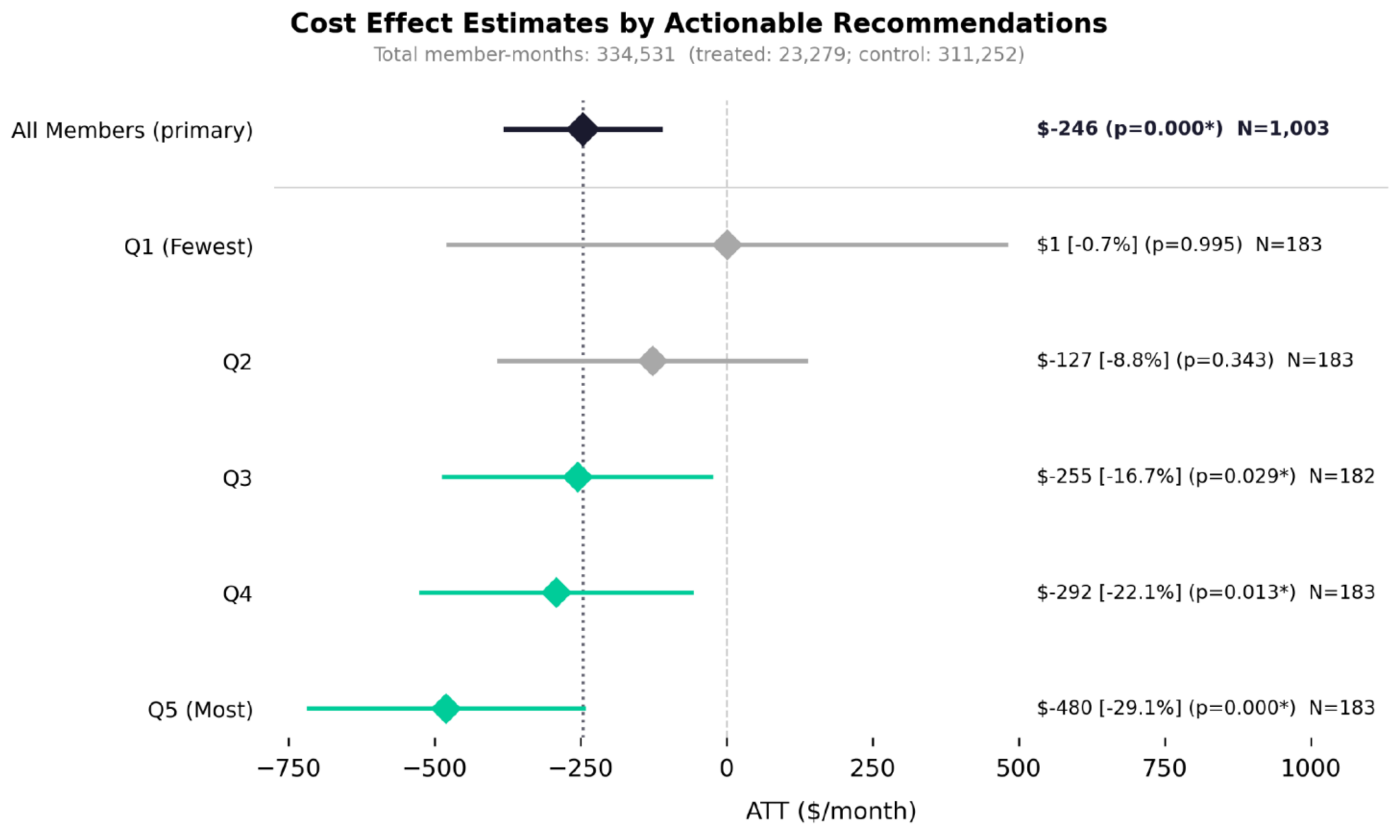

Figure A11. Forest plot of ATT estimates by number of actionable pharmacist recommendations quintiles with 95% confidence intervals; *p<0.05.

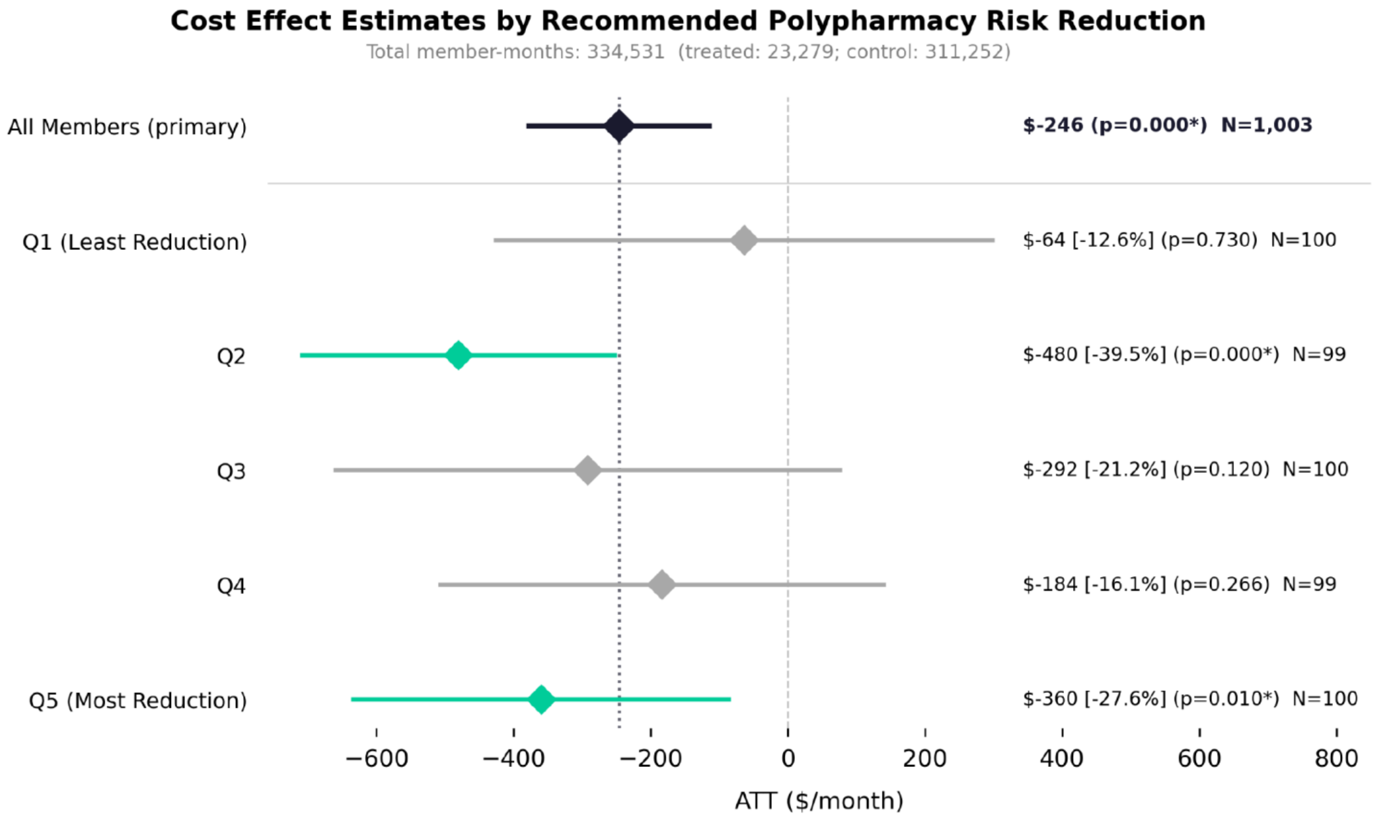

Figure A12. Forest plot of ATT estimates by total polypharmacy risk reduction magnitude quintiles (as recommended) with 95% confidence intervals; *p<0.05. Restricted to members with a recommended decrease.

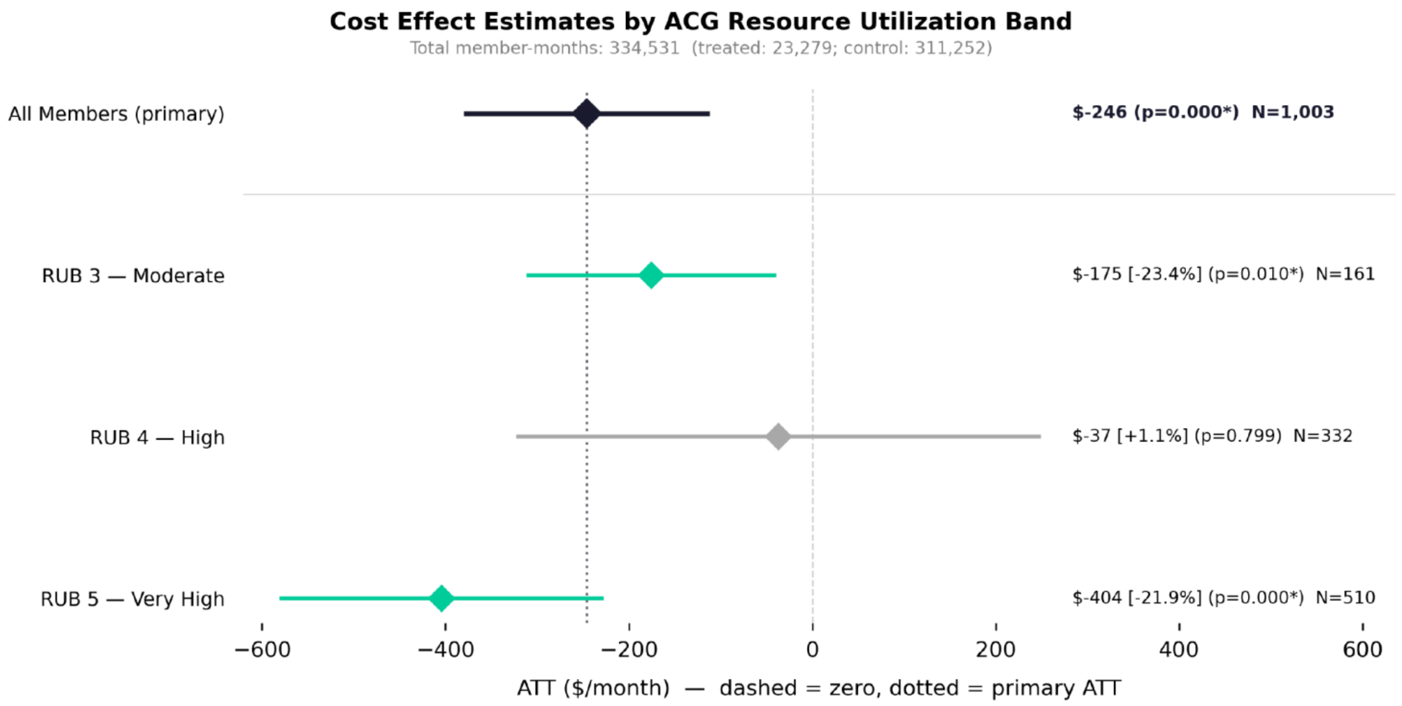

Figure A13. Forest plot of ATT estimates by ACG Resource Utilization Band (RUB 0–5). Reference line marks the primary (all-member) ATT, not zero, framing results as heterogeneity relative to the average effect. 95% confidence intervals; *p<0.05.

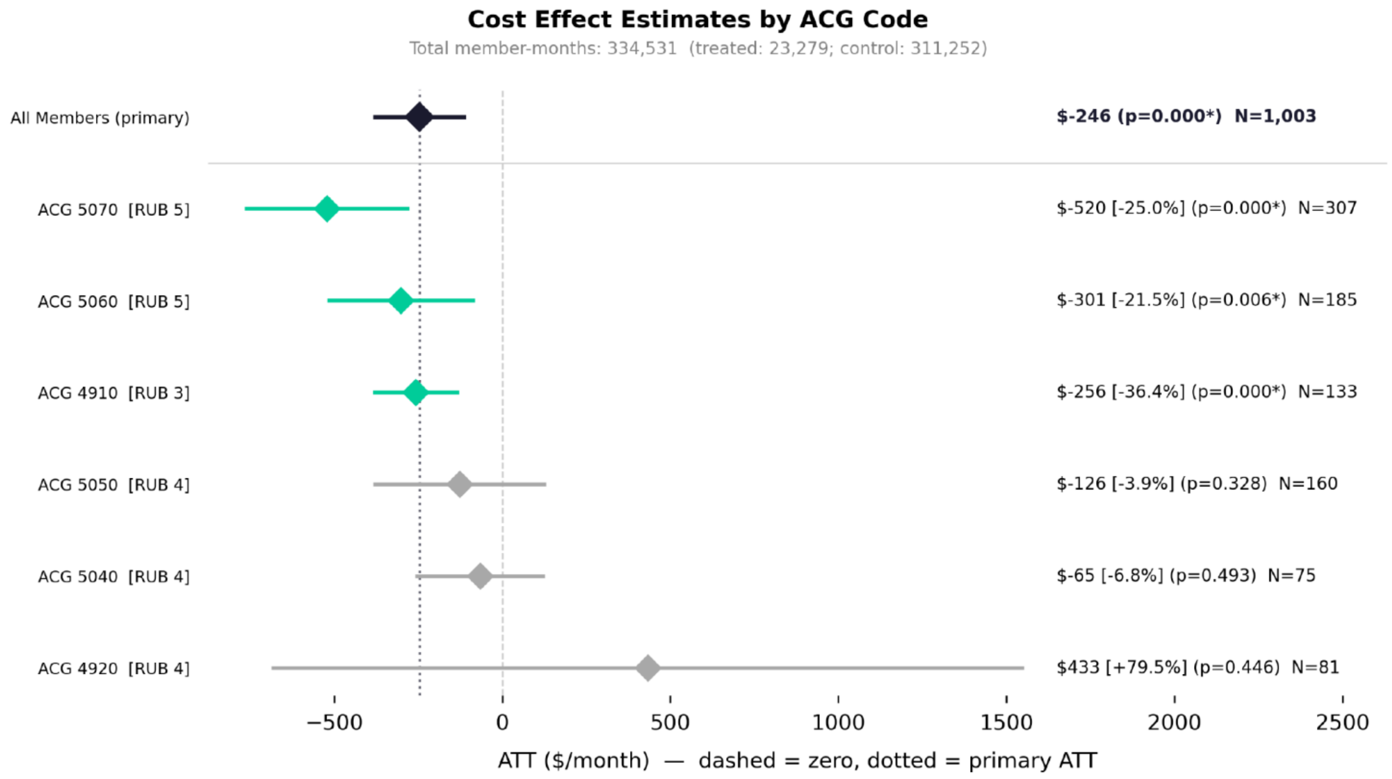

Figure A14. Forest plot of ATT estimates for qualifying ACG codes (N³30 treated members), sorted by effect magnitude. Reference line at primary ATT. *p<0.05 (uncorrected; descriptive). 95% CIs.

REGRESSION SPECIFICATIONS

Two regressions support the analysis: a propensity score model used to construct the matched cohort and the outcome model that identifies the treatment effect. Table A15 reports the propensity model refit on the matched sample (matching itself was performed by separate within-month models). A C-statistic near 0.50 is the expected result after successful matching, indicating treated and control members are no longer distinguishable on the covariates. Table A16 reports the final outcome model. Full specifications follow.

Table A15. Propensity Score Model — Multivariable Logistic Regression Predicting MTO Participation

Dependent variable: MTO participation (1 = received the intervention; 0 = control). Estimator: logistic regression. Independent variables: the 13 baseline covariates below, plus 22 calendar entry-month fixed-effect indicators (m_14–m_35) enforcing within-month matching (coefficients in source output, omitted here for brevity). All predictors are standardized (z-scored) before fitting, so coefficients and odds ratios express the change per one standard deviation of each predictor; in native units, cost variables are dollars per member-month, utilization variables are rates per member-month, and the baseline cost slope is the pre-period dollar-per-month trend.

Variable	Coefficient (log-odds)	Std. error	Odds ratio
Intercept	-2.6648	0.0335	0.070
Female	-0.0159	0.0339	0.984
Age	-0.0007	0.0354	0.999
Malignancy (any)	+0.0032	0.0353	1.003
Baseline ED visit rate	-0.0543	0.0426	0.947
Baseline IP admission rate	+0.0228	0.0655	1.023
Baseline IP day rate	+0.0343	0.0561	1.035
Baseline OP visit rate	-0.0908	0.0443	0.913
Baseline cost (PMPM)	-0.0641	0.0576	0.938
Baseline cost slope ($/mo)	-0.0411	0.0344	0.960
ACG concurrent-risk score	-0.0443	0.0462	0.957
ACG active-ingredient count	+0.1067	0.0423	1.113
ACG frailty flag	-0.0034	0.0363	0.997
ACG chronic-condition count	+0.1213	0.0497	1.129

Note: Standard errors are classical observed-information (Wald) standard errors for each logistic-regression coefficient; the Wald z-statistic is the coefficient divided by its standard error, with a normal-approximation two-sided p-value. Coefficients, standard errors, and odds ratios are on the standardized-feature scale (per 1 SD of each predictor); calendar-month fixed effects are included in the model but omitted from this table for brevity.

Model diagnostic	Value
Observations (members)	15,034 (1,003 treated; 14,031 control)
Estimator	Logistic regression (logit link)
McFadden pseudo-R^2	0.008
C-statistic (AUC)	0.567
Log-likelihood (model / null)	−3,654.31 / −3,684.21
Likelihood-ratio χ^2 (df=35)	59.8 (p = 0.006)
AIC / BIC	7,380.6 / 7,654.9
Common support (PS range)	[0.0406, 0.1184]; 100% of both arms retained

Table A16. Outcome Model — Panel Regression-Adjusted Difference-in-Differences
Dependent variable: detrended monthly medical cost ($/member-month); detrending removes calendar-time and seasonal variation estimated on control observations, with costs Winsorized at the 99.8th percentile.

Specification:

$$y_{it} = \alpha + \beta_1 \cdot Treat_i + \beta_2 \cdot Post_{it} + \beta_3 \cdot (Treat \times Post)_{it} + \Sigma_k [\gamma_k \cdot X_{k,i} + \delta_k \cdot (X_k \times Post)_{it}] + \varepsilon_{it},$$

where X_k are the 13 baseline covariates and β_3 is the ATT. Estimated by pooled member-month OLS (regression-adjusted difference-in-differences) with cluster-robust standard errors at the member level.

Term	Coefficient ($/PMPM)	Cluster-robust SE ($)	z	p (2-sided)
β_3 Treat×Post (ATT)	-246.10	67.00	-3.67	<0.001
Treat×Post (unadjusted, ref.)	-223.60	73.98	—	0.003
Female (level)	-3.41	2.00	-1.70	0.088
Female × Post	-35.48	16.53	-2.15	0.032
Age (level)	-0.94	2.11	-0.45	0.654
Age × Post	+18.15	17.11	+1.06	0.289
Malignancy (level)	-0.45	2.18	-0.21	0.838
Malignancy × Post	+82.70	18.52	+4.47	<0.001
Baseline ED rate (level)	+14.62	3.97	+3.69	<0.001
Baseline ED rate × Post	+103.35	28.90	+3.58	<0.001
Baseline IP-adm rate (level)	-29.60	8.86	-3.34	<0.001
Baseline IP-adm rate × Post	-175.77	57.61	-3.05	0.002
Baseline IP-day rate (level)	+2.98	9.33	+0.32	0.750
Baseline IP-day rate × Post	-180.16	44.63	-4.04	<0.001
Baseline OP rate (level)	-0.72	4.33	-0.17	0.868
Baseline OP rate × Post	+59.58	30.40	+1.96	0.050
Baseline PMPM (level)	+1,424.37	6.82	+208.81	<0.001
Baseline PMPM × Post	-522.23	71.20	-7.33	<0.001
Baseline cost slope (level)	+37.21	4.49	+8.30	<0.001
Baseline cost slope × Post	+140.23	34.56	+4.06	<0.001
ACG concurrent risk (level)	+3.60	3.12	+1.15	0.249
ACG concurrent risk × Post	-5.80	22.87	-0.25	0.800
ACG active-ingredient (level)	+0.90	3.21	+0.28	0.778
ACG active-ingredient × Post	+59.76	24.83	+2.41	0.016
ACG frailty (level)	-1.37	2.21	-0.62	0.535
ACG frailty × Post	+69.67	18.53	+3.76	<0.001
ACG chronic-cond (level)	+12.77	3.25	+3.93	<0.001
ACG chronic-cond × Post	+243.15	28.67	+8.48	<0.001

Note: Calendar-time and seasonal variation is removed from the dependent variable by upstream detrending on control observations (see dependent-variable definition); the outcome regression itself contains no member or calendar-time fixed-effect dummies. The intercept and the Treat and Post main effects (β_1, β_2) are estimated but omitted from the table for brevity. The reported F is the joint test of pre-period event-study leads (parallel-trends check).

**Model diagnostic**	**Value**
Dependent variable	Detrended monthly medical cost ($/member-month)
Estimator	Panel (member-month) regression-adjusted DiD (pooled OLS)
Fixed effects	None in outcome regression; calendar-time and seasonality removed via upstream detrending of the dependent variable
Standard errors	Cluster-robust at the member level
Inference	Two-sided (H_1: ATT $\neq 0$)
Observations (member-months)	334,531 (23,279 treated; 311,252 control members)
R^2 (within, covariate + interaction terms)	0.149
Detrended counterfactual (PMPM)	$1,267.68
ATT as % of counterfactual	−19.4%
Parallel-trends joint F (pre-period leads)	$F(11, 180384) = 0.299$ ($p = 0.986$)
